# Adiponectin exacerbates influenza infection in elderly individuals via IL-18

**DOI:** 10.1038/s41392-020-0141-y

**Published:** 2020-04-03

**Authors:** Youzhu Jiang, Changhua Yi, Yongxiang Yi, Qingwen Jin, Angray S. Kang, Junwei Li, Pradeep Kumar Sacitharan

**Affiliations:** 1 Department of Neurology, The Second Hospital of Nanjing, The Affiliated Hospital of Nanjing University of Chinese Medicine, #1 Zhongfu Road, Nanjing, Jiangsu Province China; 2 Department of Infectious Diseases, The Second Hospital of Nanjing, The Affiliated Hospital of Nanjing University of Chinese Medicine, #1 Zhongfu Road, Nanjing, Jiangsu Province China; 3 Public Health and Therapy Center of Nanjing, Nanjing, 211113 China; 4 Department of General Surgery, The Second Hospital of Nanjing, The Affiliated Hospital of Nanjing University of Chinese Medicine, #1 Zhongfu Road, Nanjing, Jiangsu Province China; 5 Department of Neurology, The Sir Run Run Hospital, Nanjing Medical University, #109 Longmian Avenue, Jiangning District, Nanjing, Jiangsu Province China; 6 Centre for Oral Immunobiology and Regenerative Medicine, Institute of Dentistry, Barts and the London School of Medicine and Dentistry, Queen Mary University of London, London, E1 2AT UK; 7 College of Veterinary Medicine, Qingdao Agricultural University, Qingdao, 266109 China; 8 The Institute of Ageing and Chronic Disease, University of Liverpool, Liverpool, L7 8TX UK; 9 Xi’an Jiaotong-Liverpool University, Department of Biological Sciences, #111 Ren’ai Road, Suzhou Industrial Park, Suzhou, Jiangsu Province 215123 P.R. China

**Keywords:** Ageing, Infectious diseases, Infection

**Dear Editor,**

Influenza affects humans of all ages; however, elderly people have increased susceptibility to infections and are especially predisposed to complications.^1^ A number of global efforts have increased influenza vaccination administration in elderly people to reduce complications, particularly when seasonal influenza infections peak.^2^ To date, much is known about how influenza infection and host immunity interact during pathogenesis, which has led to a number of vaccines.^3,4^ However, the impact of influenza vaccines in older people is modest.^2^ Hence, the underlying pathophysiological mechanisms responsible for the worsening of influenza infection in old age are still elusive.

Adiponectin is secreted by adipose tissue; it has insulin-sensitizing,^5^ anti-atherogenic,^6^ and anti-inflammatory properties.^7^ The hormone binds two main receptors to exert its effects: adiponectin receptor 1 (Adipor1) and adiponectin receptor 2 (Adipor2).^8^ Adiponectin signaling through the two different receptors has been shown to exert unique effects in different disease states and tissues.^8,9^ Interestingly, adiponectin levels are elevated in elderly individuals, showing negative correlations with several age- and obesity-related metabolic disturbances.^10^ However, the role of adiponectin and its signaling during infections in the elderly population have not been investigated.

Our initial observation was that adiponectin levels were elevated in bronchoaveolar lavage fluid (BALF) obtained from old patients with confirmed influenza infection (Fig. 1a). We also observed mRNA and protein expression of adiponectin in cultured lung tissue cells from old patients with confirmed influenza infection that was greater than that of matched and young control cells (Fig. 1b, c and Supplementary Fig. 1a). Only IL-18 mRNA expression was increased in aged lung tissue with confirmed influenza infection compared to age-matched and young control tissue (Fig. 1d). An increase in adiponectin and IL-18 concentrations in the sera of either young or old patients with or without influenza infection was not detected (Supplementary Fig. 1b, c). We next treated isolated human lung cells with recombinant adiponectin to test whether IL-18 levels were directly correlated with increased adiponectin levels in lung tissue cells. IL-18 mRNA expression was elevated after treatment of aged lung tissue cells with recombinant adiponectin, and it was further increased in aged lung tissue with influenza infection compared to that of control cells (Fig. 1e). At this stage, we wanted to investigate whether a loss or decrease of adiponectin could decrease the worsening of influenza. Interestingly, we noticed that only aged *Adipoq*^*−/−*^ mice displayed reduced disease outcomes following influenza infection compared to those of control mice (Fig. 1f, g and Supplementary Fig. 1c-d). In addition, only *Adipoq*^*−/−*^ old mice with influenza infection showed a decrease in IL-18 mRNA and protein expression (Supplementary Fig. 1e, f). There was no change in IL-18 concentrations in the sera of young or old WT and *Adipoq*^*−/−*^ mice (Supplementary Fig. 1g, h). We also established that neutralizing adiponectin could act as a therapeutic agent that could attenuate the worsening of influenza infection in old age. Only old WT mice with influenza infection showed protection and reduced disease outcomes after anti-adiponectin antibody treatment (Fig. 1h, i and Supplementary Fig. 2a, b). Compared to control mice, these mice also displayed decreased IL-18 mRNA (lung tissue; Supplementary Fig. 2c) or protein (BALF; Supplementary Fig. 2d) expression following influenza infection.

**Fig. 1:**
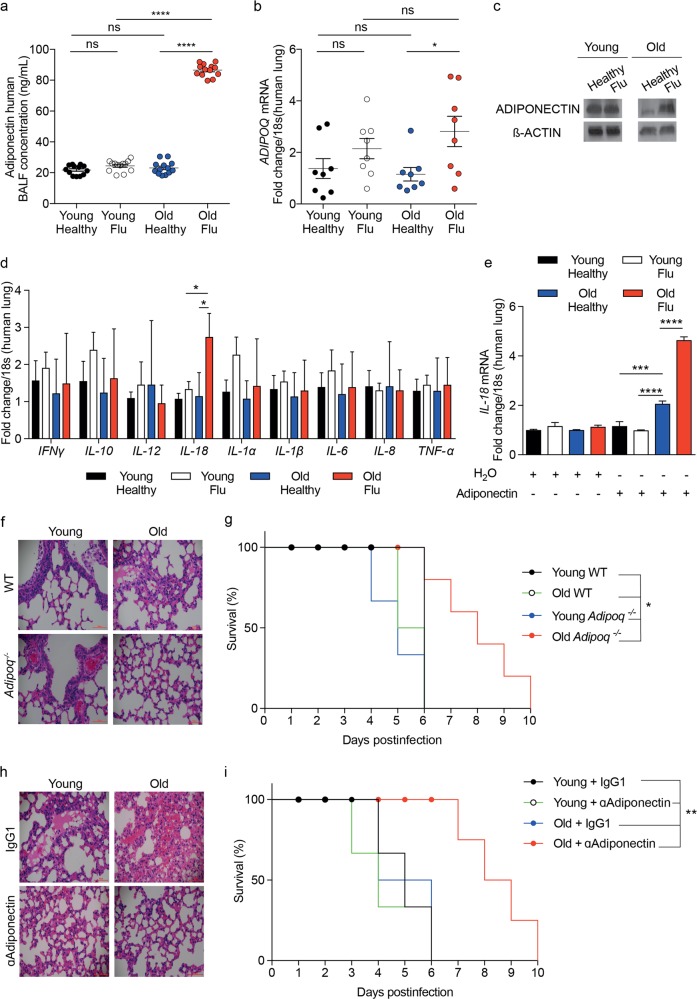
Adiponectin exacerbates influenza infection in old age via IL-18. **a** Adiponectin concentration in human BALF samples obtained from young (18–35) or old (60–74) patients with or without influenza (*n* = 8–13). **b** Gene and (**c**) Protein expression of adiponectin from cultured lung samples obtained from young (18–35 old) or old (60–74 old) patients with or without influenza. **d** Gene expression of cytokines from lung biopsies obtained from young (18–35 old) or old (60–74 old) patients with or without influenza (*n* = 8). **e** IL-18 mRNA expression in cultured lung samples obtained from young (18–35 old) or old (60–74 old) patients with or without influenza treated with either the vehicle control (H_2_O) or human rAdiponectin (3 μg/ml) for 24 h (*n* = 3). **f** H&E staining of the lungs (**g**) survival rate of influenza-infected (10×MLD_50_ H1N1 influenza virus) WT or *Adipoq*^*−/−*^ young (3 months to 9 months old) and old (20 months to 24 months old) mice (*n* = 6–12). **h** H&E staining of the lungs (**i**) survival rate of influenza-infected (10×MLD_50_ H1N1 influenza virus) WT young (3 months to 9 months old) and old (20 months to 24 months old) mice treated with either the control (IgG1: 50 µg per mouse daily) or with mouse adiponectin antibody (50 µg per mouse daily) until the final timepoints (*n* = 6–12). All RT-qPCR gene expression levels were normalized to the endogenous level of 18 s. All data are expressed as the mean ± S.E.M. of *n* observations. A Student’s unpaired *t* test or ANOVA with Tukey’s comparison were used for statistical analysis. Survival curves were compared using log rank Mantel–Cox curve comparison, and groups were compared to either old *Adipoq*^*−/−*^ mice or old WT mice + rAdiponectin in their respective graphs. NS = non–significant. *p* < 0.05, *p* < 0.01, *p* < 0.001 or *p* < 0.0001 are represented in figures as *, **, *** or ****, respectively

We also investigated which main adiponectin receptor was responsible for the increase in IL-18 levels during influenza infection in old age. We observed that only old *Adipor1*^*−/−*^ mice displayed decreased disease outcomes post influenza infection compared to that of control mice (Supplementary Fig. 3a-d). IL-18 mRNA (lung tissue; Supplementary Fig. 3e) or protein (BALF; Supplementary Fig. 3f) expression was only decreased in old *Adipor1*^*−/−*^ mice with influenza infection compared to control mice. In contrast, *Adipor2*^*−/−*^ mice did not show any change in disease outcomes (Supplementary Fig. 4a-d) or IL-18 mRNA (lung tissue; Supplementary Fig. 4e) or protein (BALF; Supplementary Fig. 4f) expression following influenza infection compared to that of control mice. We also treated old WT and IL-18^*−*/*−*^ mice with rAdiponectin to test whether the worsening of influenza infection in old age caused by adiponectin was IL-18 dependent. We observed that only IL-18^*−*/*−*^ mice with or without rAdiponectin had increased survival following influenza infection compared to those of control mice (Supplementary Fig. 5a-d). In addition, old wild-type mice treated with rAdiponectin showed an increase in IL-18 mRNA (lung tissue; Supplementary Fig. 5e) or protein (BALF; Supplementary Fig. 5f) expression following influenza infection compared to that of control mice. Finally, we observed decreased protein expression and activity of intercellular proteins involved in the activation cascade of IL-18 in human lung samples (Supplementary Fig. 6f, g). However, we did not determine a detailed immune mechanism of why neutralizing adiponectin helps stop the worsening of influenza infection in elderly individuals, which is a limitation of our study. Future studies should concentrate on investigating the response of CD8^+^ T cells in this context and other immune mediators, such as interferon stimulated genes.

In summary, our data point to a new detrimental role for the hormone adiponectin in aged lung tissue during influenza infection. Adiponectin signaling seems to have no effect on healthy and infected young models or on old healthy models, which shows the potential of targeting this novel signaling axis only in elderly patients during periods of influenza infection. We also further defined adiponectin signaling in aged mice with infection and showed that the direct action of the hormone is dependent on Adiopr1-IL-18 signaling. Adiponectin may prove to be a new therapeutic target for intervention and reducing the severity of influenza infections in elderly people.

## Supplementary information

SUPPLEMENTAL MATERIAL
