# Supplementary material for: Adiponectin exacerbates influenza infection in elderly individuals via IL-18
Source: Signal Transduct Target Ther. 2020 Apr 3;5:32. doi: 10.1038/s41392-020-0141-y (PMC7118100; doi:10.1038/s41392-020-0141-y)
Supplement: Supplementary file 1 — SUPPLEMENTAL MATERIAL [file 41392_2020_141_MOESM1_ESM.docx]

Supplementary Materials for

Adiponectin exacerbates influenza infection in elderly individuals via IL-18.

Youzhu Jiang^1^*, Changhua Yi^2,3^*, Yongxiang Yi^4^, Qingwen Jin^5^, Angray S Kang^6^, Junwei Li^7^# and Pradeep Kumar Sacitharan^8,9^#.

*Equal contributors

#Corresponding authors:

Dr. Pradeep Kumar Sacitharan ; E-mail: PK.Sacitharan@xjtlu.edu.cn

**This PDF file includes:**

Materials and Methods

Figures. S1 to S6

Tables S1

**Material and Methods**

**Human samples**

The ethics committee of The Second Hospital of Nanjing reviewed and approved this study. Biopsies, BALF and blood samples from young (18-35 old) or old (60-74 old) patients diagnosed with influenza by lower respiratory sampling and RT (real time)-qPCR (quantitative polymerase chain reaction) verification^1,2^ (induced sputum, endotracheal aspiration or bronchoscopy) were used. Healthy samples were obtained from donors without respiratory diseases during autopsies. Full informed written consent from patients and donors was obtained and approved by The Second Hospital of Nanjing. All experiments and tissue collection were in full compliance with the Declaration of Helsinki. Bronchoscopies^3^, BALF^4^ and blood collections^5^ were conducted as previously described.

**Human lung tissue cell culture and treatments**

Human lung biopsy tissues were washed with phosphate buffer saline (PBS; Sigma-Aldrich) and cut into small pieces. Thereafter tissues were enzymatically digested for 45 min in RPMI media (Sigma-Aldrich) with DNase I (Sigma-Aldrich) and Collagenase (Roche) at 37°C with stirring. After enzymatic digestion, cold RPMI with 10% FCS (fetal calf serum; Biological Industries), 4.1 mM L-Glutamine (Sigma-Aldrich) and 1% penicillin/streptomycin (Sigma-Aldrich) was added to stop the enzymatic process. Cell suspension was passed through a 70 μm filter and washed with media and PBS with 2% FCS. Red blood cells were lysed using 5 ml of ammonium-chloride-potassium lysing buffer for 5 min on ice and washed with PBS 2% FCS. Cell suspension was resuspended in PBS 2% FCS. Cells were then plated in RPMI with 10% FCS, 4.1 mM L-Glutamine and 1% penicillin/streptomycin at 37°C until confluence. For some experiments human cells were serum-starved overnight (10% FCS Media) then treated with either the vehicle control of H_2_O or human r(recombinant)Adiponectin (3 μg/ml; 450-24; PepROTech) for 24 h^6^. For other experiments human cells were serum-starved overnight (10% FCS Media) then treated with human rAdiponectin (3 μg/ml) and the control of IgG1 (1 µg/ml; Sigma-Aldrich) or human adiponectin antibody (1 µg/ml; Abcam) for 24 h.

**Mice**

Murine experiments were approved and conducted with the guidance of the ethics committee at the College of Veterinary Medicine, Qingdao Agricultural University. Mice used in experiments were on a C57BL/6N background, provided *ad libitum* water and a standard laboratory chow diet and maintained 25 ± 1 °C, humidity at 55 ± 5% and 12-h light–dark cycles. *Adipoq^-/-^*, *Adipor1^-/-^*, *Adipor2^-/-^* and *IL-18^-/-^* mice were originally obtained from Jackson Laboratories. Wild-type (WT) mice were from a C57BL/6N background.

**Influenza model**

H1N1 (PR8) influenza virus was propagated in 9-day-old specific pathogen-free (SPF) embryonated chicken eggs (Melia, Beijing). After a 72-hour incubation at 37°C, allantoic fluids were harvested and tested by hemagglutination assay with 1% SPF chicken red blood cells and stored at -80°C. Madin-Darby canine kidney (MDCK) (ATCC) cells were cultured in modified Eagle's medium (MEM) supplemented with gentamycin, sodium pyruvate and 5% fetal bovine serum. Viral titers by calculating the 50% egg infectious dose (EID_50_)/mL and 50% mouse lethal dose (MLD_50_ ) were determined by the method of Reed and Muench^7,8^. Young (3 months to 9 months old) and old mice (20 months to 24 months old) were in were anesthetized and inoculated intranasally with 50 μl of 10×MLD_50_ influenza virus. Control mice were treated with PBS in the same way. Mice were monitored three times daily after virus infection. The mice were humanely euthanized when one of the following termination criteria were met: body weight loss ≥30%, body temperature ±2°C, cardiac/respiratory rate ±50%, and sign of severe pneumonia. Persons handling live viruses were protected with HEPA-filtered respirator units. Lung samples^9^, BAL^9^ and blood collections were conducted as previously described at the end timepoint. BALF was collected at day 4 post infection.

***In vivo* treatments**

For some experiments after the inoculation of H1N1 influenza virus mice were treated with the control of saline or with mouse rAdiponectin (1 mg/kg daily; 315-26; PepROTech)^10^ until end timepoints. For other experiments after the inoculation of H1N1 influenza virus mice treated with the control of IgG1 (50 µg per mouse daily; Sigma-Aldrich) or with mouse adiponectin antibody (50 µg per mouse daily; ab3455; Abcam)^11,12^ until end timepoints.

**Histology**

Murine lung specimens were collected at end time points, fixed in 10% neutral buffered formalin, and embedded in paraffin wax. Five-micrometer-thick sections were stained with hematoxylin and eosin (H&E).

**Titration of infectious virus**

At the end timepoints lungs of mice were dissected, weighed and further homogenized with 1 ml PBS as 1g/ml (w/v). The supernatant was aliquoted and stored at -80°C. Titration (plaque forming unit per mL, PFU/mL) of infectious virus in lung homogenates was carried out in MDCK cells by standard plaque forming assay, and the viral titers was calculated using the Spearman–Karber method.

**RNA analysis**

Total RNA was isolated from cell cultures or lung samples using RNeasy Mini Kit (Qiagen) as per manufacturer’s instructions. Reverse transcription was carried out by PCR with Expand High Fidelity DNA Polymerase (Roche). Thereafter, RT-qPCR was carried out to analyse relative gene expression by the ^∆∆^Ct method using 18s as an endogenous control gene. Details of TaqMan primers used in experiments can be found in Supplementary table 1.

**Protein analysis**

Total protein was extracted from cell cultures using a lysis buffer containing radioimmunoprecipitation assay buffer (Sigma-Aldrich) and a phosphatase and protease inhibitor cocktail (Sigma-Aldrich). Protein levels of samples were determined by bicinchoninic acid assay (Thermo Fisher Scientific). Samples were probed overnight with primary antibody: Adiponectin (~30 kDa; ab22554; Abcam), β-actin (~42 kDa; A2228; Sigma-Aldrich), p-p38 (phospho Y182; ~41 kDa; ab47363; Abcam), p38 (~42 kDa ; ab27986; Abcam), p-IκBα (phospho S36; ~35 kDa; ab133462; Abcam), p-IκBα (~36 kDa; 07-1483; Sigma-Aldrich) or IL-18 (~25 kDa; AF2548; R&D systems). Densitometric analysis was conducted using ImageJ.

**ELISA**

Adiponectin (human: DRP300; R&D systems) and IL-18 (human: ab215539; Abcam; mouse: ab216165; Abcam) concentrations were measured via ELISA. Assays were conducted as per the manufacturer’s instructions.

**Statistical analysis**

Experiments were statistically analysed utilising the Students unpaired t-test for parametric data with independent groups compared with their specific controls or time-matched controls. One-way analysis of variance (ANOVA) with the Bonferroni post hoc test or ANOVA with Tukeys comparison test was used to compare 3 or more groups. All data are expressed as mean ± standard error of mean (S.E.M) of *n* observations. A significant difference was accepted when p< 0.05, p< 0.01, p<0.001 or p< 0.0001 represented in all tables and Figures as *, **, *** or **** respectively. NS= non–significant. Data analysis was performed using GraphPad Prism® 5.0 (GraphPad Software).

**
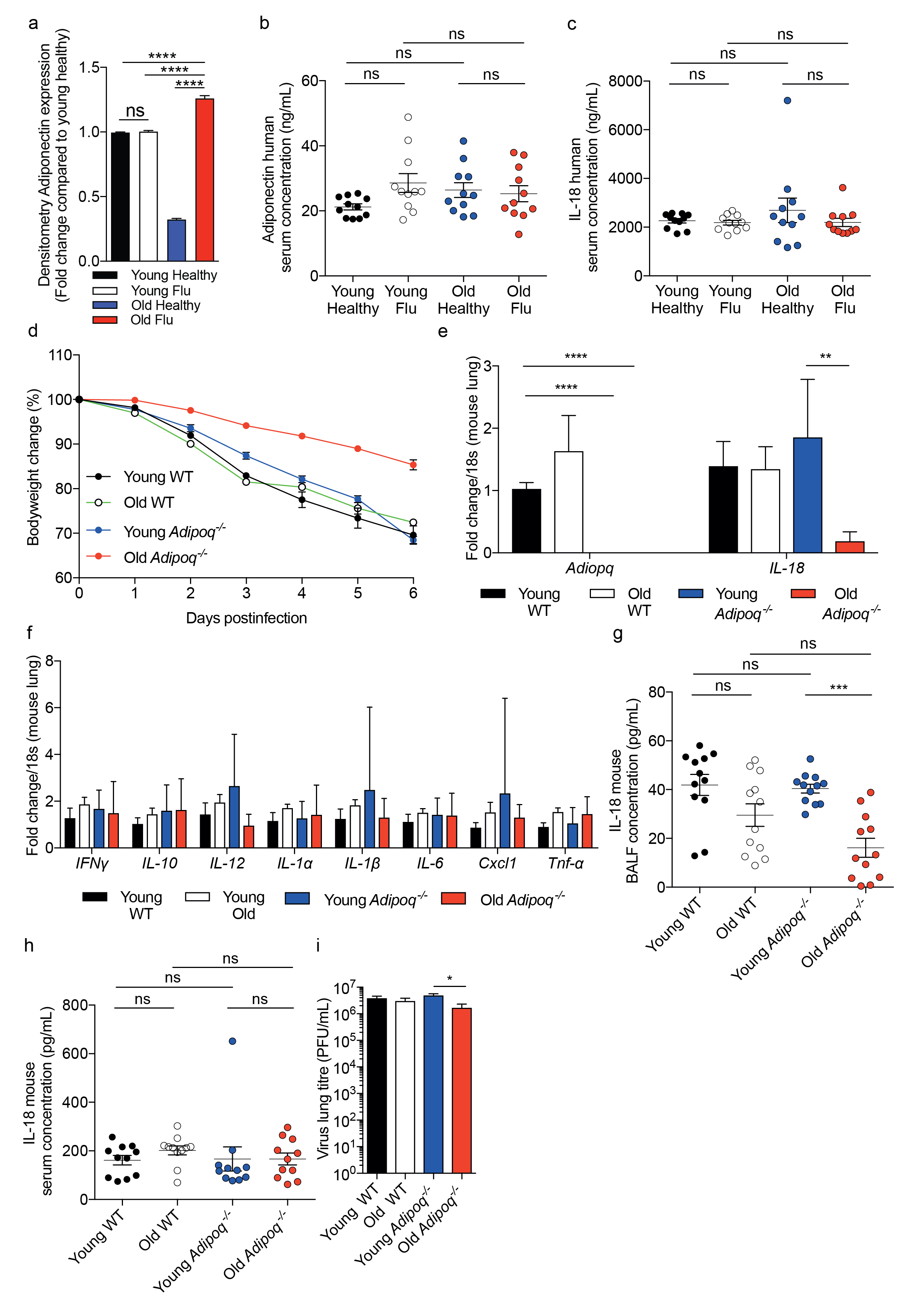
**

**Supplementary Fig. 1: Adiponectin exacerbates influenza infection in old age via IL-18.**

**a,** quantification of protein expression of Adiponectin from cultured lung samples obtained from young (18-35 old) or old (60-74 old) patients with or without influenza (*n*=4). **b,** Adiponectin and **c,** IL-18 concentration in human sera obtained from young (18-35) or old (60-74) patients with or without influenza (*n*=11). **d,** bodyweight change **e,** end-point mRNA lung tissue expression of *Adipoq* and *IL-18* from influenza-infected (10×MLD_50_ H1N1 influenza virus) WT or *Adipoq^-/-^* young (3 months to 9 months old) and old (20 months to 24 months old) mice (*n*=6-12). **f,** Gene expression of cytokines from lung tissue obtained from influenza-infected (10×MLD_50_ H1N1 influenza virus) WT or *Adipoq^-/-^* young (3 months to 9 months old) and old (20 months to 24 months old) mice (*n*=8). **g,** BALF and **h,** sera concentration of IL-18 **g,** virus titre (day 4 post infection) in WT or *Adipoq^-/-^* young (3 months to 9 months old) and old (20 months to 24 months old) mice infected with influenza (10×MLD_50_ H1N1 influenza virus) (*n*=11). All RT-qPCR gene expressions were normalised to the endogenous level of 18s. All data are expressed as mean ± S.E.M of *n* observations. ANOVA with Tukeys comparison was used for statistical analysis. NS= non–significant. p< 0.05, p< 0.01, p< 0.001 or p< 0.0001 represented in figures as *, **, *** or **** respectively.

**
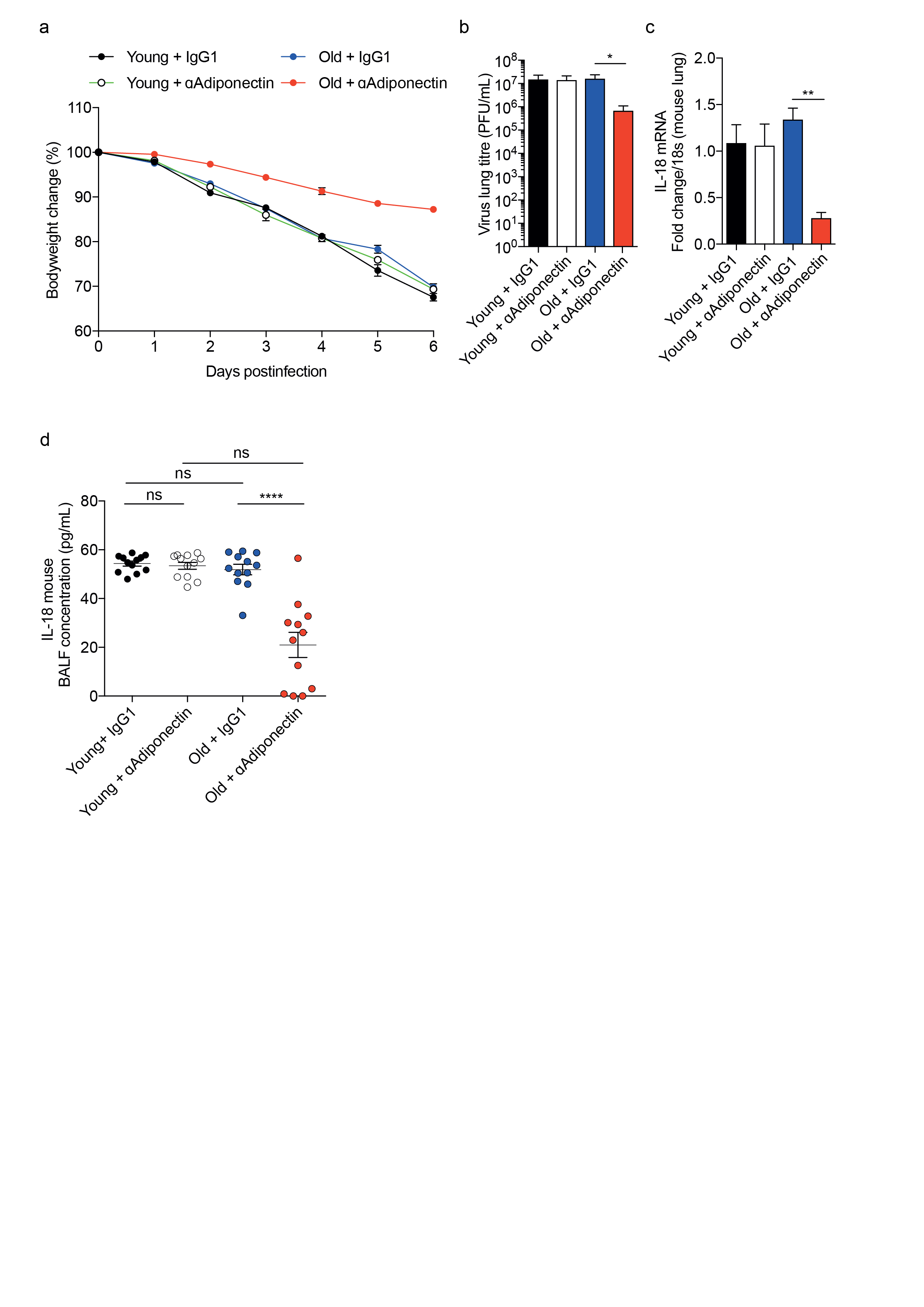
**

**Supplementary Fig. 2: Neutralising adiponectin attenuates the exacerbation of influenza infection in old age.**

**a,** bodyweight change **b,** virus titre (day 4 post infection) **c,** end-point mRNA lung tissue expression of *IL-18* from influenza-infected (10×MLD_50_ H1N1 influenza virus) WT young (3 months to 9 months old) and old (20 months to 24 months old) mice treated with either the control of IgG1 (50 µg per mouse daily) or with mouse adiponectin antibody (50 µg per mouse daily) until end timepoints (*n*=6-12). **d,** IL-18 concentration in young (3 months to 9 months old) and old (20 months to 24 months old) mice infected with influenza (10×MLD_50_ H1N1 influenza virus) and treated with either the control of IgG1 (50 µg per mouse daily) or with mouse adiponectin antibody (50 µg per mouse daily) until end timepoints (*n*=12). All RT-qPCR gene expressions were normalised to the endogenous level of 18s. All data are expressed as mean ± S.E.M of *n* observations. Students unpaired t-test or ANOVA with Tukeys comparison were used for statistical analysis. NS= non–significant. p< 0.05, p< 0.01 or p< 0.0001 represented in figures as *, ** or **** respectively.

**
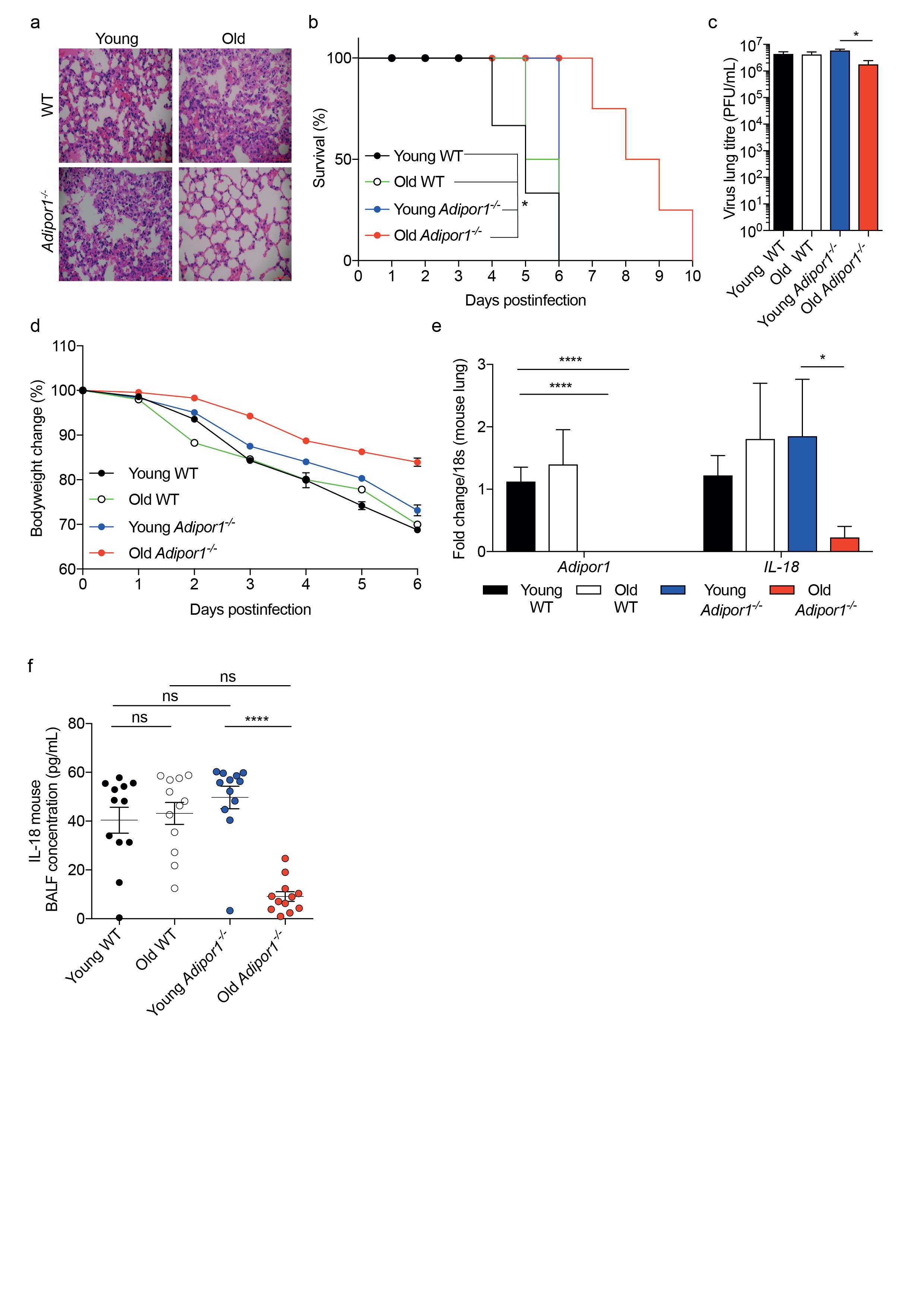
**

**Supplementary Fig. 3: Adiponectin-IL-18 induced exacerbation of influenza infection in old age is mediated by Adipor1.**

**a,** H&E staining of the lungs **b,** survival rate **c,** virus titre (day 4 post infection) **d,** bodyweight change **e,** end-point mRNA lung tissue expression of *Adipor1* and *IL-18* from influenza-infected (10×MLD_50_ H1N1 influenza virus) WT or *Adipor1^-/-^* young (3 months to 9 months old) and old (20 months to 24 months old) mice (*n*=6-12). **f,** IL-18 concentration in *Adipor1^-/-^* young (3 months to 9 months old) and old (20 months to 24 months old) mice infected with influenza (10×MLD_50_ H1N1 influenza virus) (*n*=12). All RT-qPCR gene expressions were normalised to the endogenous level of 18s. All data are expressed as mean ± S.E.M of *n* observations. Students unpaired t-test or ANOVA with Tukeys comparison were used for statistical analysis. Survival curves were compared using log rank Mantel-Cox curve comparison, groups were compared to old *Adipoq1^-/-^* mice. NS= non–significant. p< 0.05 or p< 0.0001 represented in figures as * or **** respectively.

**
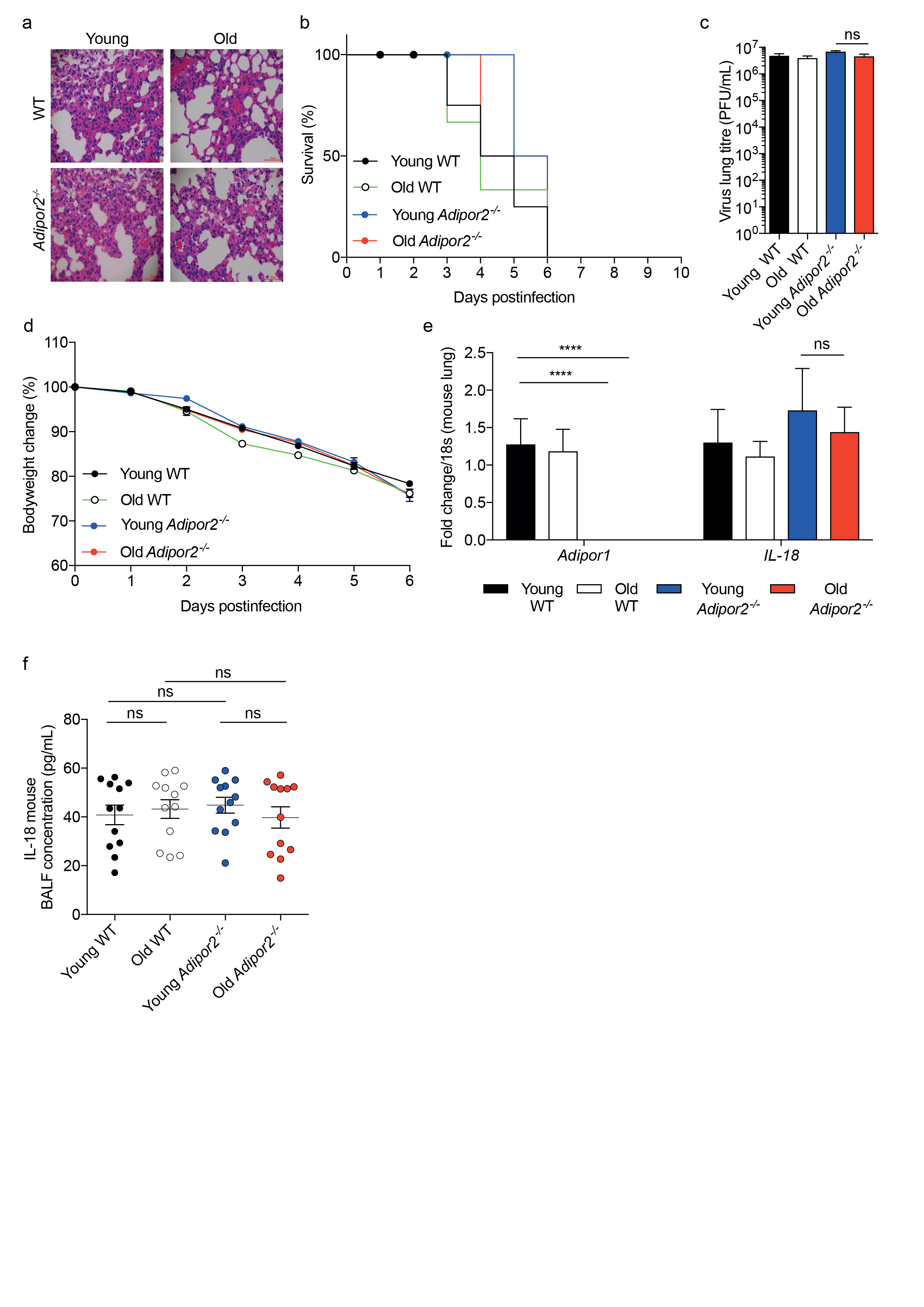
**

**Supplementary Fig. 4: Adiponectin-IL-18 induced exacerbation of influenza infection in old age is not mediated by Adipor2.**

**a,** H&E staining of the lungs **b,** survival rate **c,** virus titre (day 4 post infection) **d,** bodyweight change **e,** end-point mRNA lung tissue expression of *Adipor2* and *IL-18* from influenza-infected (10×MLD_50_ H1N1 influenza virus) WT or *Adipor2^-/-^* young (3 months to 9 months old) and old (20 months to 24 months old) mice (*n*=6-12). **f,** IL-18 concentration in *Adipor2^-/-^* young (3 months to 9 months old) and old (20 months to 24 months old) mice infected with influenza (10×MLD_50_ H1N1 influenza virus) (*n*=12). All RT-qPCR gene expressions were normalised to the endogenous level of 18s. All data are expressed as mean ± S.E.M of *n* observations. Students unpaired t-test or ANOVA with Tukeys comparison were used for statistical analysis. Survival curves were compared using log rank Mantel-Cox curve comparison, groups were compared to old *Adipoq2^-/-^* mice (NS difference was found). NS= non–significant. p< 0.05 or p< 0.0001 represented in figures as * or **** respectively.

**
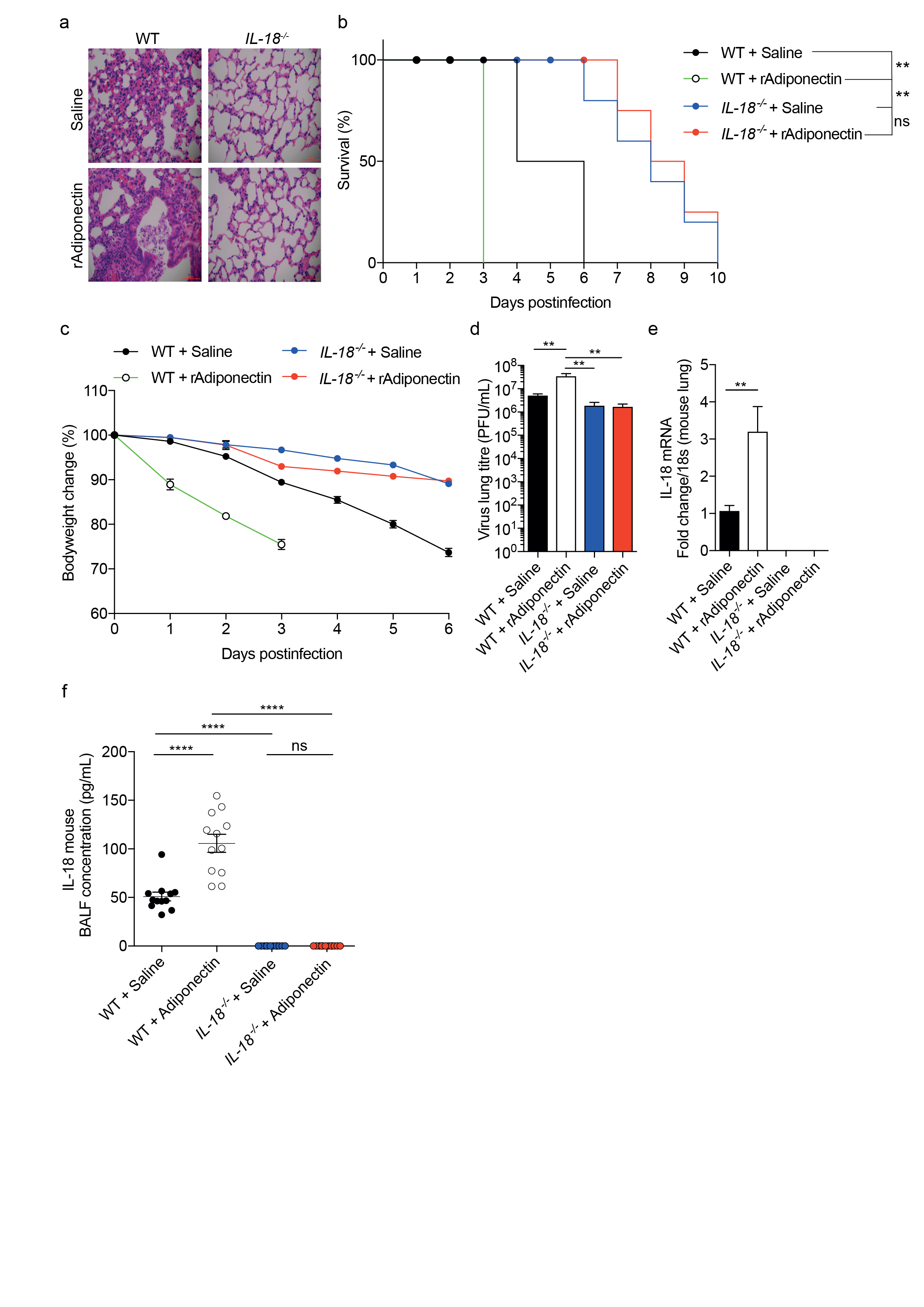
**

**Supplementary Fig. 5: Adiponectin induced exacerbation of influenza infection in old age is nullified in IL-18^-/-^ mice.**

**a,** H&E staining of the lungs **b,** survival rate **c,** bodyweight change **d,** virus titre (day 4 post infection) **e,** end-point mRNA lung tissue expression of *IL-18* from influenza-infected (10×MLD_50_ H1N1 influenza virus) old (20 months to 24 months old) WT or *IL-18^-/-^* mice treated with either the control of saline or with mouse rAdiponectin (1 mg/kg daily) (*n*=6-12). **f,** IL-18 concentration in old (20 months to 24 months old) *IL-18^-/-^* mice infected with influenza (10×MLD_50_ H1N1 influenza virus) and treated with either the control of saline or with mouse rAdiponectin (1 mg/kg daily) (*n*=12). All RT-qPCR gene expressions were normalised to the endogenous level of 18s. All data are expressed as mean ± S.E.M of *n* observations. Students unpaired t-test or ANOVA with Tukeys comparison were used for statistical analysis. Survival curves were compared using log rank Mantel-Cox curve comparison, groups were compared to old *IL-18^-/-^* mice + rAdiponectin. NS= non–significant. p< 0.01 or p< 0.0001 represented in figures as ** or **** respectively.

**
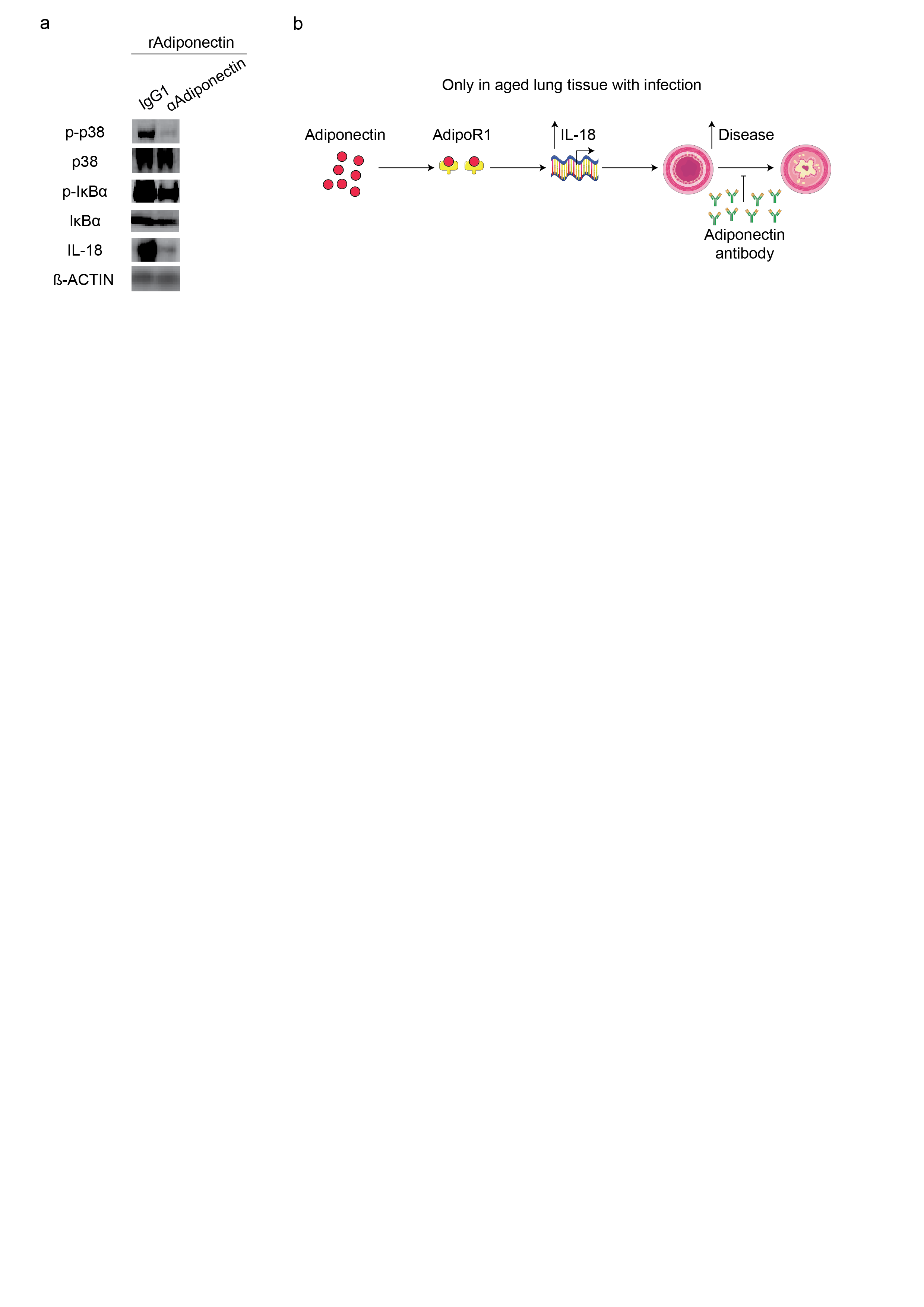
**

**Supplementary Fig. 6: Neutralising adiponectin attenuates the exacerbation of influenza infection in old age. a,** protein expression of IL-18 signaling cascade in cultured lung samples obtained from old (60-74 old) patients. Cells were treated with human rAdiponectin (3 μg/ml) and the control of IgG1 (1 µg/ml;) or human adiponectin antibody (1 µg/ml) for 24 h. **b,** Adiponectin signaling via Adipor1 increases the expression of IL-18 to exacerbate influenza infection only in aged lung tissue. This exacerbation can be inhibited by neutralising adiponectin.

**Supplementary Table 1:** TaqMan probes used in RT-qPCR experiments.

| **Gene** | **Human** | **Murine** |
| --- | --- | --- |
| *18S* | Hs03003631_g1 | Mm02601776_g1 |
| *Adipoq* | Hs00605917_m1 | Mm00456425_m1 |
| *Adipor1* | Hs00360422_m1 | Mm01291334_mH |
| *Adipor2* | Hs00226105_m1 | Mm01184032_m1 |
| *IFNγ (IFNG)* | Hs00989291_m1 | Mm01168134_m1 |
| *Il-1α* | Hs00174092_m1 | Mm00439620_m1 |
| *Il-1β* | Hs01555410_m1 | Mm00434228_m1 |
| *Il-10* | Hs00961622_m1 | Mm01288386_m1 |
| *Il-12* | Hs01073447_m1 | Mm01288989_m1 |
| *Il-18* | Hs01038788_m1 | Mm00434226_m1 |
| *Il-6* | Hs00174131_m1 | Mm00446190_m1 |
| *Il-8 (CXCL8)* | Hs00174103_m1 | - |
| *Cxcl1* | - | Mm04207460_m1 |
| *TNF-α* | Hs00174128_m1 | Mm00443258_m1 |

**Supplementary references**

1. Kumar, Y. *et al.* Serum Proteome and Cytokine Analysis in a Longitudinal Cohort of Adults with Primary Dengue Infection Reveals Predictive Markers of DHF. *PLoS Negl. Trop. Dis.* **6**, e1887 (2012).

2. Wang, R., Sheng, Z.-M. & Taubenberger, J. K. Detection of Novel (Swine Origin) H1N1 Influenza A Virus by Quantitative Real-Time Reverse Transcription-PCR. *J. Clin. Microbiol.* **47**, 2675 LP – 2677 (2009).

3. Koslow, M. *et al.* The role of bacterial culture by bronchoscopy in patients with lung cancer: a prospective study. *J. Thorac. Dis.* **9**, 5300–5305 (2017).

4. Chen, J., Ryu, S., Gharib, S. A., Goodlett, D. R. & Schnapp, L. M. Exploration of the normal human bronchoalveolar lavage fluid proteome. *Proteomics - Clin. Appl.* (2008). doi:10.1002/prca.200780006

5. Boura, P. *et al.* The prognostic value of serum and bronchoalveolar lavage levels of adiponectin in advanced non-small-cell lung cancer. *Lung Cancer Manag.* (2017). doi:10.2217/lmt-2016-0018

6. Sharma, K. *et al.* Adiponectin regulates albuminuria and podocyte function in mice. *J. Clin. Invest.* **118**, 1645–1656 (2008).

7. Li, J., Cai, H., Liu, Q. & Guo, D. Molecular and pathological characterization of two H5N1 avian influenza viruses isolated from wild ducks. *Virus Genes* (2008). doi:10.1007/s11262-008-0245-x

8. Li, J. *et al.* Single mutation at the amino acid position 627 of PB2 that leads to increased virulence of an H5N1 avian influenza virus during adaptation in mice can be compensated by multiple mutations at other sites of PB2. *Virus Res.* (2009). doi:10.1016/j.virusres.2009.04.008

9. Kawaguchi, A. *et al.* Impacts of allergic airway inflammation on lung pathology in a mouse model of influenza A virus infection. *PLoS One* **12**, e0173008 (2017).

10. Liu, Z. *et al.* Adiponectin reduces ER stress-induced apoptosis through PPARα transcriptional regulation of ATF2 in mouse adipose. *Cell Death Dis.* (2016). doi:10.1038/cddis.2016.388

11. Liu, J. *et al.* Adiponectin is critical in determining susceptibility to depressive behaviors and has antidepressant-like activity. *Proc. Natl. Acad. Sci. U. S. A.* (2012). doi:10.1073/pnas.1202835109

12. Dai, Y., Pang, J., Gong, H., Fan, W. & Zhang, T. M. Roles and tissue source of adiponectin involved in lifestyle modifications. *Journals Gerontol. - Ser. A Biol. Sci. Med. Sci.* (2013). doi:10.1093/gerona/gls131
